# Joint Semi-Blind Self-Interference Cancellation and Equalisation Processes in 5G QC-LDPC-Encoded Short-Packet Full-Duplex Transmissions

**DOI:** 10.3390/s22062204

**Published:** 2022-03-11

**Authors:** Bao Quoc Vuong, Roland Gautier, Hien Quang Ta, Lap Luat Nguyen, Anthony Fiche, Mélanie Marazin

**Affiliations:** 1 Univ Brest, CNRS, Lab-STICC, CS 93837, 6 Avenue Le Gorgeu, CEDEX 3, 29238 Brest, France; roland.gautier@univ-brest.fr (R.G.); anthony.fiche@univ-brest.fr (A.F.); melanie.marazin@univ-brest.fr (M.M.); 2 School of Electrical Engineering, International University, Ho Chi Minh City 700000, Vietnam; tqhien@hcmiu.edu.vn (H.Q.T.); nlluat@hcmiu.edu.vn (L.L.N.); 3 Vietnam National University, Ho Chi Minh City 700000, Vietnam

**Keywords:** full-duplex, digital self-interference cancellation, 5G LDPC codes, short-packet transmission, semi-blind channel estimation, recursive least-squares algorithm, sum of product algorithm

## Abstract

The paper proposes a joint semi-blind algorithm for simultaneously cancelling the self-interference component and estimating the propagation channel in 5G Quasi-Cyclic Low-Density Parity-Check (QC-LDPC)-encoded short-packet Full-Duplex (FD) transmissions. To avoid the effect of channel estimation processes when using short-packet transmissions, this semi-blind algorithm was developed by taking into account only a small number (four at least) pilot symbols, which was integrated with the intended information sequence and used for the feedback loop of the estimation of the channels. The results showed that this semi-blind algorithm not only achieved nearly optimal performance, but also significantly reduced the processing time and computational complexity. This semi-blind algorithm can also improve the performances of the Mean-Squared Error (MSE) and Bit Error Rate (BER). The results of this study highlight the potential efficiency of this joint semi-blind iterative algorithm for 5G and Beyond and/or practical IoT transmission scenarios.

## 1. Introduction

The Internet of Things (IoT) has become an integral part of human life with various applications in industry, manufacturing, agriculture, healthcare, etc. The development of 5G wireless communications enables many new devices to communicate and to be able to make autonomous decisions by deploying diverse technologies and connecting massive devices [1,2,3]. The 5G network supports and targets two main services for IoT applications such as ultra-Reliable Low-Latency Communications (uRLLC) and massive Machine-Type Communications (mMTC) [4]. uRLLC is a new class of performance communications that focuses strictly on the highest possible reliability and enables latency as low as 1 ms, since it concentrates on supporting mission-critical applications such as intelligent transportation and industry automation [2,5]. mMTC focuses mainly on efficiently transmitting a low data amount intermittently to and from devices that require wide area coverage and a long battery life, which can be up to thousands of devices such as wearable or smart applications and sensors in the IoT [6]. In order to be efficient, short-length transmissions or short-packet transmissions should be used certainly for both uRLLC and mMTC in their applications [1]. Furthermore, compared to the infinite packet transmission system, short-length packet transmission is considered as a foundation of Physical Layer Security (PLS) problems in 5G and IoT applications to ensure the robustness of artificial noise or self-jamming techniques [7]. Last but not least, as 5G has gone to the final stage, 6G has received much interest from the research community because it is targeted to support more diversified applications. Therefore, both the uRLLC and mMTC techniques should be explored deeply in order to fully support short-packet communications, not only to provide an efficient data transmission, but also to ensure communication reliability [8,9].

Since the deployment of 5G will lead to a network expansion by offering a platform for connecting a large number of IoT devices, it requires new fundamental solutions for sharing the spectrum efficiently. Full-Duplex (FD) transmission, simultaneously transmitting and receiving data through the same resource (time and frequency), is a promising technique for 5G and Beyond wireless networks because it can “theoretically” double the spectral efficiency, compared to the traditional Half-Duplex (HD) method such as Time Division Duplex (TDD) or Frequency Division Duplex (FDD) [10]. However, to achieve the doubled spectral efficiency in practice for this FD transmission, the Self-Interference (SI) component must be cancelled close to the noise floor level, which is not an easy task due to the high power, which may reach up to 120 dB in the real communication networks and then strongly disturb the reception behaviour [11]. Therefore, SI cancellation would play the most critical role in implementing FD communication systems in both academia [12,13] and industry [14,15]. Nevertheless, the use of an FD transmission with good control of SI can provide many advantages to modern wireless communications systems, both in terms of spectral efficiency, as well as reliability and security through the use of self-jamming techniques [16]. In order to cancel the SI component to a reasonable level, many techniques have been proposed such as the passive technique with Radio Frequency (RF) cancellation (beamforming, antenna decoupling or isolation, cross-polarisation, etc.) [17,18,19] or active techniques with analogue cancellation [20,21] (analogue filter, etc.) and digital cancellation [22].

Although the SI component’s cancellation has already been implemented at the RF and analogue level, in order to cancel it to an acceptable level, a large amount of the residual SI component still needs to be continuously cancelled in the digital domain. Therefore, a Digital Self-Interference Cancellation (DSIC) process has been applied to estimate the residual SI and cancel it from the received signal. DSIC is usually based on adaptive filtering with the knowledge of the SI signal provided by the transmitter, and the intended channel can be estimated by using an equaliser with blind or semi-blind estimations [23,24,25]. In recent years, the authors in [26,27] proposed joint algorithm channel estimation for SI cancellation and signal detection in FD transmission. However, the results were not satisfactory in short-packet transmissions because the systems require many data symbols to obtain a good second-order statistics of the received signal. Therefore, the constraints of time-, bandwidth-, and power-efficient approaches for short-packet transmission in FD transmission have to be considered carefully. Indeed, a potential technique for channel estimation and data detection in short-packet FD transmission is to consider semi-blind channel estimation, which is the concatenation between the known pilot symbols and the information symbols in order to form a transmitted sequence [28,29,30,31]. For instance, the authors in [29] proposed an iterative semi-blind receiver with Carrier Frequency Offset (CFO) for uRLLC in short-packet FD transmission systems. Moreover, a semi-blind FD Amplify-and-Forward (AF) relay system with adaptive SI processing assisted by Independent Component Analysis (ICA) was proposed in order to improve the low latency and high reliability in IoT communications [30]. Furthermore, a new Semi-blind Minimum Mean-Squared Error (S-MMSE) technique was also proposed to further suppress the residual SI power in FD mmWave massive MIMO systems [31]. Their proposed algorithm was used to overcome the problem of ergodic capacity and outage capacity, as well as the length of pilot symbols, which are the biggest challenges in short-packet transmissions. Moreover, there still exist some limitations in FD short-packet transmissions such as the high error in the SI channel estimation [29] and the high latency of the decoding process, i.e., in the 5G Quasi-Cyclic Low-Density Parity Check (QC-LDPC) decoder [32,33], because the system needs to use many decoding iterations to reach the convergence or saturation level. Therefore, many concerns of researchers focus on these challenges of the FD short-frame transmission.

In this paper, a semi-blind algorithm is proposed for joint iterative SI cancellation and intended channel estimation in 5G QC-LDPC-encoded FD short-packet transmissions in the digital domain, by taking into account only a small number (four at least) of pilot symbols and the feedback of the estimate of the channel to achieve a nearly optimal performance and efficient use in practical scenarios.

Throughout the paper, the performance evaluation of the proposed algorithm is based on four metrics: Mean-Squared Error (MSE), Bit-Error-Rate (BER), processing time, and computational complexity.

The contributions of this paper can be summarised as follows:

- We propose a joint iterative *semi-blind* SI cancellation and channel estimation in 5G QC-LDPC-encoded short-packet FD transmissions;
- We characterise the out-performance of the system with the proposed algorithm compared to the conventional algorithm. In particular, this *semi-blind* technique can significantly improve the performances of the MSE and BER, while requiring only the addition of a few pilot symbols for the channel estimation feedback processes;
- We point out that the time consumption and computational complexity of the proposed algorithm are lower than the conventional algorithm, which is suitable for IoT applications and green communications.

The remainder of this paper is organised as follows. Section 2 describes briefly the system model of FD transmissions with the conventional DSIC algorithm and the 5G QC-LDPC channel coding scheme. Section 3 proposes the joint iterative *semi-blind* channel estimation and decoding algorithm. Then, the numerical results and comparisons with the conventional algorithm and the comparison of the processing time and computational complexity of all schemes are presented in Section 4. After that, some highlights and conclusions are discussed in Section 5. Finally, the potential works in the near future are presented in Section 6. The notations in this paper are summarised in Table 1.

## 2. Conventional SB _DSICED3 _W/OF Scheme

### 2.1. System Model

**Figure 1:**
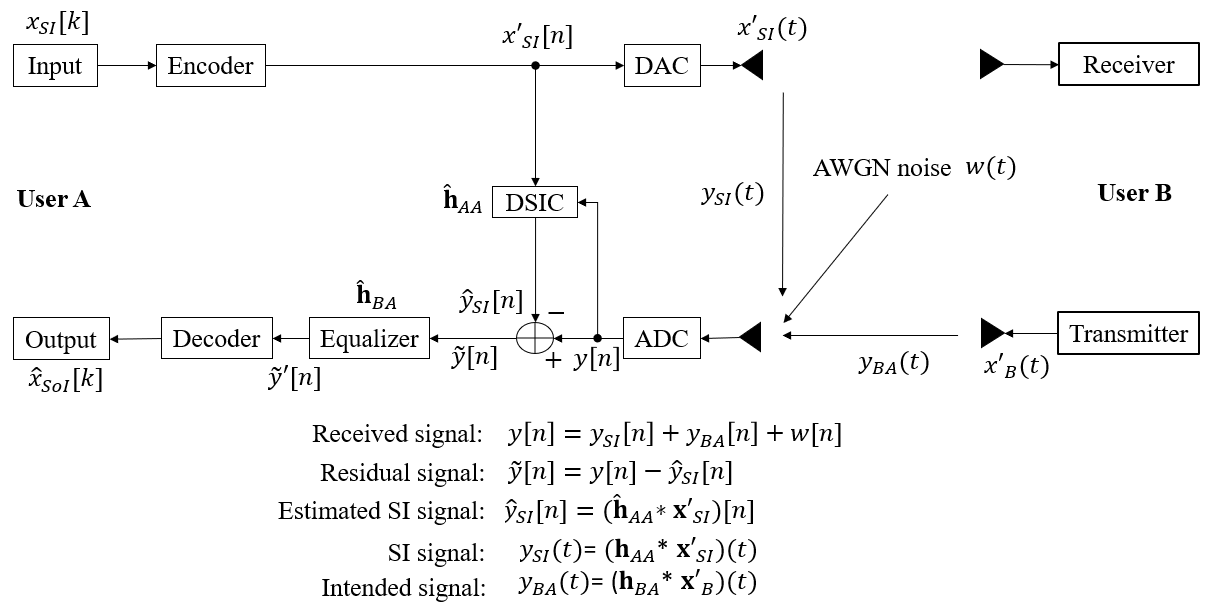
SISO FD transmission with the SB_DSICED3_W/OF process.

Let us consider a short-packet transmission model between two users, A and B, which are equipped with two antennas for simultaneously transmitting and receiving information in FD modes, as shown in Figure 1. At both User A and B’s transceivers, the channel coding scheme is based on 5G QC-LDPC codes for both uplink and downlink short-packet transmissions [34]. We assumed that the intended channel gains between two users and the SI channel gains at User A itself are denoted by $h_{BA}$ and $h_{AA}$, respectively, which are i.i.d. complex Gaussian random variables with CN(0,1). In FD transmissions, the SI channel normally consists of two components: Line-of-Sight (LoS) and Non Line-of-Sight (NLoS). By using passive cancellation and analogue cancellation techniques, the LoS component can be cancelled significantly while the reflections remain [22]. Therefore, in the digital domain, the SI channel can be modelled as a Rayleigh fading distribution.

In FD transmissions, the channel estimation and decoding performance will be the same for both Users A and B because of the symmetric characteristic. Without loss of generality, we considered that the receiver side of User A and its received signal in the time domain can be given by:

(1) $$\begin{array}{cc} y(t) & =y_{SI}\left(t\right)+y_{BA}\left(t\right)+w\left(t\right), \end{array}$$

where $y_{SI}\left(t\right)$ is the self-interference component consisting of the SI signal $x_{SI}^{\prime }$ (the transmitted signal at User A is denoted by $x_{SI}^{\prime }$ rather than $x_{A}^{\prime }$ in order to distinguish the SI and the intended signal) propagating through the SI channel $h_{AA}$ and $y_{BA}\left(t\right)$ is the received component consisting of the intended signal $x_{B}^{\prime }$ passing through the propagation channel $h_{BA}$. Therefore, Equation (1) can be expressed as:

(2) $$\begin{array}{cc} y(t) & =\left(h_{AA}*x_{SI}^{\prime }\right)\left(t\right)+\left(h_{BA}*x_{B}^{\prime }\right)\left(t\right)+w\left(t\right). \end{array}$$

The operator (∗) denotes the convolution, and w(t) is the complex Gaussian noise with $\mathrm{CN}(0,\sigma_{w}^{2})$.

Then, the received summation signal is passed to the Analogue-to-Digital Converter (ADC) process to be converted to the discrete time domain signal, y[n]. Here, in order to overcome the effects of residual quantisation noise error, the bit resolution and voltage dynamic range of Digital-to-Analogue Converter (DAC)/ADC devices should be chosen carefully and highly enough, which was implemented in [35], or the oversampling method should be applied if a low-bit resolution of ADC devices is used [36]. In this paper, we further assumed that the impacts of DAC/ADC, other hardware impairments on the SI cancellation, and the problem of the synchronisation process were not considered (which is outside of the scope of this study, but essential in practice). After that, the DSIC process is applied to obtain the estimated SI channel ${\hat{h}}_{AA}$ by using an adaptive filter with the Recursive Least-Squares (RLS) algorithm, and its forgetting factor λ should be chosen between 0.9 and 1 [37]. The RLS algorithm was chosen because it has a faster convergence and better performance in the DSIC process compared to other algorithms such as the Least Mean Squares (LMS) or Normalised Least Mean Squares (NLMS) [38,39,40]. A reference signal $x_{SI}^{\prime }\left[n\right]$ from the transmitter side of User A can be used to cancel the SI component to obtain:

(3) $$\tilde{y}\left[n\right]=y\left[n\right]-{\hat{y}}_{SI}\left[n\right]=y\left[n\right]-\left({\hat{h}}_{AA}*x_{SI}^{\prime }\right)\left[n\right].$$

Then, an equaliser using the RLS algorithm is applied with the assistance of pilot symbols, which are integrated into the information message modulated sequence in the encoder process at the transmitting side of User B, in order to estimate the intended channel and obtain the equalised signal. After channel estimation and the equaliser processes, these pilot symbols are eliminated and the binary output ${\hat{x}}_{SoI}\left[k\right]$ of the signal of interest can be obtained from the equalised signal ${\tilde{y}}^{\prime }\left[n\right]$ via demodulation, de-interleaving, and decoding in the decoder process.

This transmission model is called the conventional algorithm, and we name it as the *SB_DSICED3_W/OF* scheme, for the Semi-Blind Digital Self-Interference Cancellation, Equalisation, Demodulation, De-interleaving, and Decoding Without Feedback scheme. Next, we briefly introduce the channel coding scheme named the 5G QC-LDPC coding, encoder, and decoder processes, which is used to form the transmitted signal $x_{SI}^{\prime }\left(t\right)$ and $x_{B}^{\prime }\left(t\right)$ from Users A and B, respectively.

### 2.2. 5G QC-LDPC Coding, Encoder, and Decoder Processes

**Figure 2:**
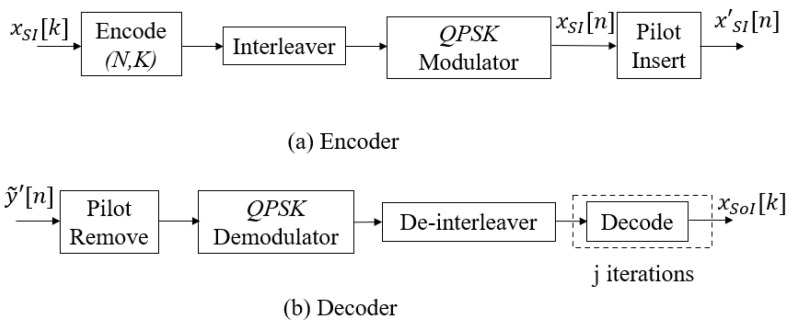
Encoder and decoder processes.

In 1962, R. Gallager introduced the first classical LDPC codes [41], and it was rediscovered again by MacKay in the late 1990s [42]. In 2018, the 3rd Generation Partnership Project (3GPP) chose QC-LDPC codes as the standard codes for 5G networks and applications based on the properties and characteristics of classical LDPC codes, especially for a short-length frame with lower processing throughput for uRLLC and the mMTC or massive Machine-to-Machine (mM2M) communications [32,34,43,44,45]. Moreover, the authors in [46,47,48] showed that 5G QC-LDPC codes are also an optimised design for short-packet transmission because of the low error floor and high-speed transmission. The construction of the encoder and decoder processes are described in Figure 2. At the transmitting side, the binary input signal $x_{SI}\left[k\right]$, where k∈[1,K], is encoded by using the (N,K) 5G LDPC encoding process to form a codeword with length *N*. The encoding technique between the exponent parity check matrix H and the information bit sequence is based on the Gauss–Jordan elimination algorithm [49], where *N* and *K* denote the codeword length and information length, respectively. Then, the obtained codeword is interleaved and modulated by using the Quadrature Phase Shift Keying (QPSK) modulator with the modulation order M=4, in order to form the modulated signal $x_{SI}\left[n\right]$, where n∈[1,E] and $E=N/log_{2}\left(M\right)$. Then, the βE known pilot symbols, where β is the pilot symbol coefficient and (0<β≤1), are added to the message sequence after the modulation process in order to form the transmitted signal $x_{SI}^{\prime }\left[n\right]$ with a length of $E^{\prime }=\left(1+\beta \right)E$. Finally, this signal is passed to the DAC process to convert to the continuous time signal $x_{SI}^{\prime }\left(t\right)$. The encoder process for the signal of interest $x_{B}\left[k\right]$ to form transmitted intended signal $x_{B}^{\prime }\left(t\right)$ is similar to that of the SI signal $x_{SI}\left[k\right]$.

At the receiver side, the residual signal after the DSIC process $\tilde{y}\left[n\right]$ is passed through the equalisation process with the RLS algorithm to estimate the intended channel and obtain the equalised signal ${\tilde{y}}^{\prime }\left[n\right]$. Then, the pilot symbols are removed from this signal before going to the QPSK demodulator and de-interleaver processes to form the Log Likelihood Ratio (LLR) belief sequence. Then, this LLR sequence is used for decoding and decisions. To obtain the estimated binary input signal ${\hat{x}}_{SoI}\left[k\right]$ of Node B, the Sum Product Algorithm (SPA) [50,51,52] is applied at Node A, which is the message passing between the check nodes and the symbol nodes for guessing the transmitted bits from each other at each iteration *j* until it reaches the maximum number of decoding interactions $j_{max}$.

## 3. Proposed Joint Iterative Semi-Blind Scheme Version

In this section, we propose a joint iterative semi-blind channel estimation and decoding scheme version, named JISB_DSICED3 for Joint Iterative Semi-Blind Digital Self-Interference Cancellation, Equalisation, Demodulation, De-interleaving, and Decoding scheme.

**Figure 3:**
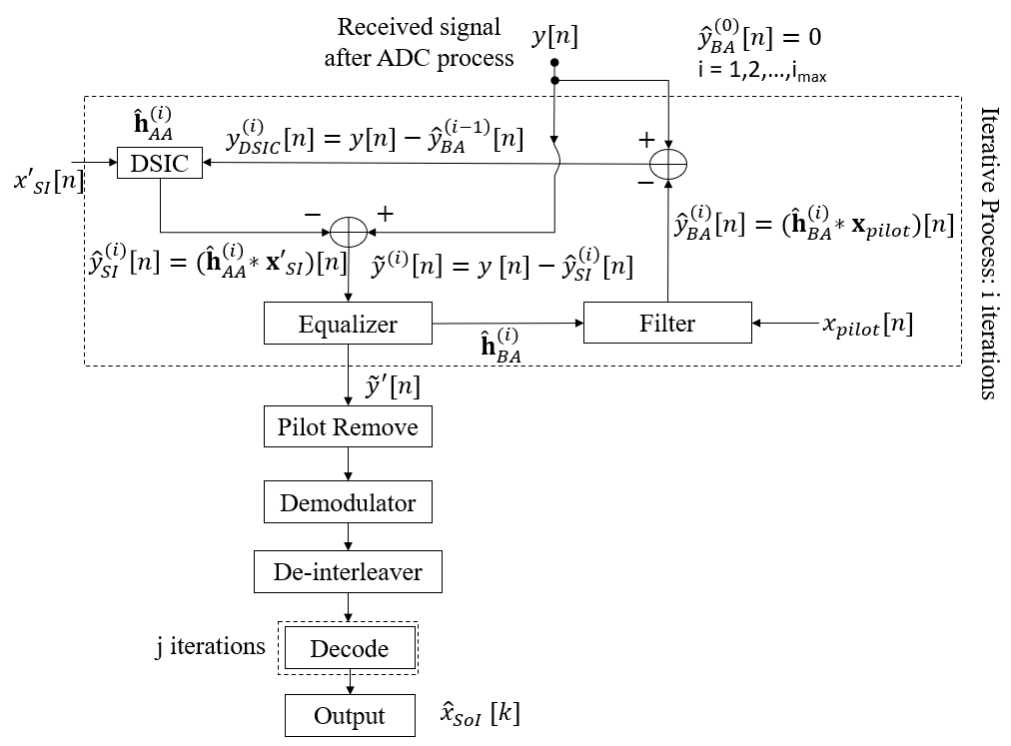
SISO FD transmission with the proposed JISB_DSICED3 process.

**Figure 4:**
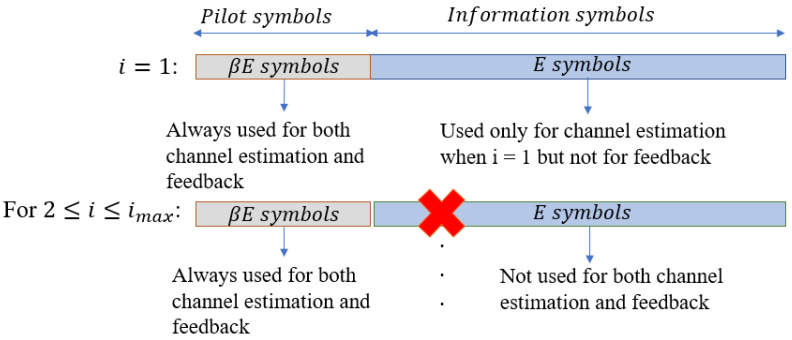
Graphical presentation for the joint iterative semi-blind scheme version.

The process of the semi-blind algorithm is shown in Figure 3, in which the proposed scheme was developed on the principle that the processes of SI cancellation and the intended channel estimation can benefit from each other via the feedback loop after each joint iteration *i*, where $i\in [1,i_{max}]$. We emphasise that, different from the iteration *j* performing the iteration decoding in the conventional algorithm in Section 2.1, the iteration *i* in the proposed algorithm is for the joint SI cancellation and the intended propagation channel estimation via feedback. Based on the knowledge of pilot symbols transmitted by Node B, the system does not need to perform the temporary decoding and encoding for feedback. Indeed, it only performs the feedback loop by these pilot symbols $x_{pilot}$. Denote βE, where β is the pilot symbol coefficient and (0<β≤1), as the number of pilot symbols that is added to the *E* modulated symbols of the information signal sequence after the encoding, interleaver, and modulation process to form $E^{\prime }=\left(1+\beta \right)E$ symbols for a transmission frame. It should be noted that for i=1 (first iteration), a first SI cancellation and intended channel estimation is performed for all $E^{\prime }$ symbols, which is used to avoid a significant number of errors and obtain a good level of convergence when starting the process of the iterative algorithm. For the remaining iterations, i.e., $i\in [2,i_{max}]$, the system only performs the feedback loop by the known pilot symbols $x_{pilot}$ with the length of βE. After the system completely performs the joint iterative process, i.e., $i=i_{max}$, the estimations of the SI channel and intended channel are used to fully cancel the SI component and achieve the equalised signal, respectively. Then, these pilot symbols are removed from the equalised signal ${\tilde{y}}^{\prime }\left[n\right]$, and the system continues to perform the decoder process by the de-modulation, de-interleaver, and decoding processes, in order to achieve the intended message sequence $x_{SoI}$. It should be noted that performing many *j* iterations in the decoding process will increase the latency and complexity because of the high computational complexity in the SPA decoding process [53]. Therefore, when we achieve the best channel estimation ($i=i_{max}$), the proposed scheme will only consider one iteration of decoding ($j_{max}=1$) in the SPA decoding algorithm to obtain a good result, because if we include more decoding iterations *j*, this does not improve the performance significantly. The graphical presentation of the proposed joint iterative semi-blind SI cancellation and equalisation processes algorithm is shown in Figure 4, and the proposed iterative algorithm with the three main steps is summarised in Algorithm 1.

**Algorithm 1:** Iterative part of the proposed joint iterative semi-blind scheme.

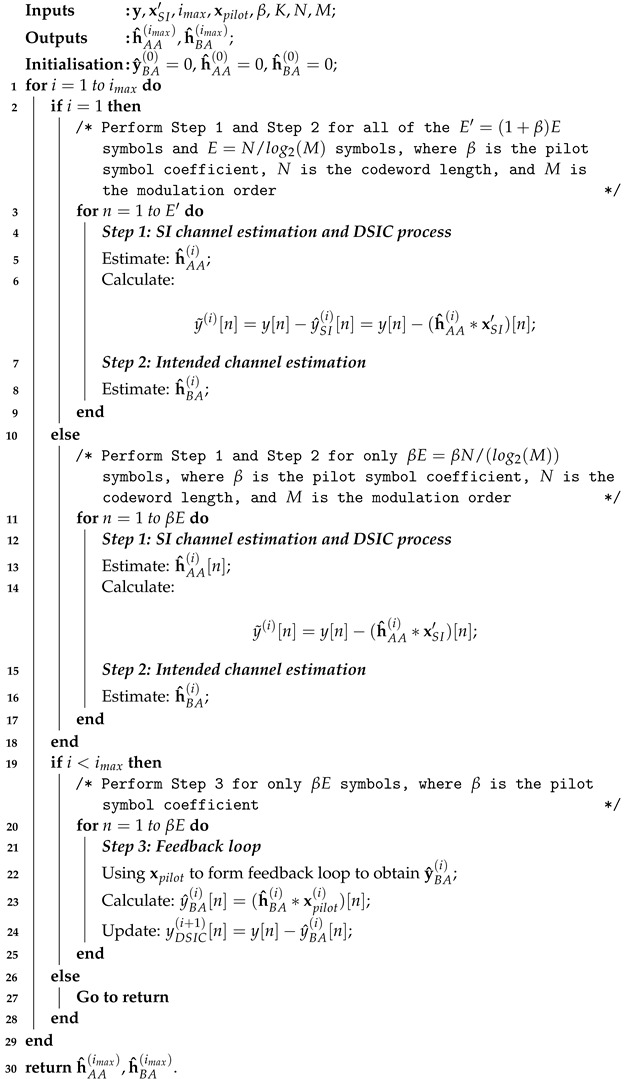



## 4. Simulation Results and Discussion

In this paper, the performances of the proposed scheme, compared with the conventional scheme SB_DSICED3_W/OF, are illustrated in terms of the MSE, BER, and processing time by using Monte Carlo simulations in MATLAB. For the 5G QC-LDPC codes, the base graph matrix **BG2** was chosen for all simulations. The index of the iterations for the joint iterative decoding and the 5G QC-LDPC decoding is denoted as *i* and *j*, respectively. Many simulations with various channel models and the number of channel taps (ranging from 3–8 taps) were implemented. However, the varying of the channel models did not significantly change the performance results. Due to the limitation and not to overload the paper, all of the configurations and figures are not provided for reasons of readability of the paper and better understanding. Therefore, the SI channel was fixed with three taps, while the intended propagation channel was fixed with four taps according to the ITU–R Pedestrian test environment channel model [54]. These channels were generated independently in each transmission frame. The simulation parameters are summarised in Table 2. It was also noticed that the semi-blind without feedback scheme SB_DSICED3_W/OF needed up to 20 iterations ($j_{max}=20$) to converge and to reach the saturation floor, while the feedback scheme JISB_DSICED3 required only four iterations ($i_{max}=4$) to reach that floor.

### 4.1. MSE Performances: JISB _DSICED3 versus SB _DSICED3 _W/OF and the Best Performance Scheme

In this subsection, we also introduce, as a benchmark to characterise the optimality of the semi-blind scheme JISB_DSICED3 in terms of the MSE and for the performance comparison, a particular scheme called the Best Performance Scheme (BPS) corresponding to a lower bound (but not realistic in practice) using the proposed JISB_DSICED3 considering that all $E^{\prime }=\left(1+\beta \right)E$ are known. Therefore, in this limit case, all the intended *E* symbols are also known and not only the pilots symbols for both channels’ estimation and feedback. The MSE of the SI channel and the intended channel are respectively given by [26]:

(4) $${\mathrm{MSE}}_{SI}=|h_{AA}-{\hat{h}}_{AA}^{\left(i\right)}{|}^{2},$$

(5) $${\mathrm{MSE}}_{BA}=|h_{BA}-{\hat{h}}_{BA}^{\left(i\right)}{|}^{2}.$$

**Figure 5:**
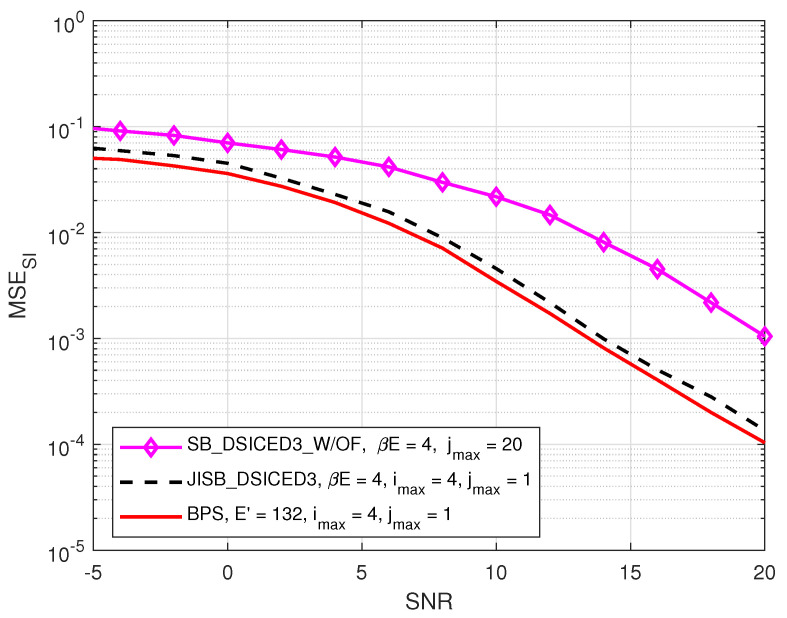
$MSE_{SI}$ versus SNR, $p_{B}/\sigma_{w}^{2}$; R=1/2, $i_{max}=4$, $j_{max}=20$, $p_{SI}/\sigma_{w}^{2}=30$ dB, βE=4 symbols, and E=128 symbols.

**Figure 6:**
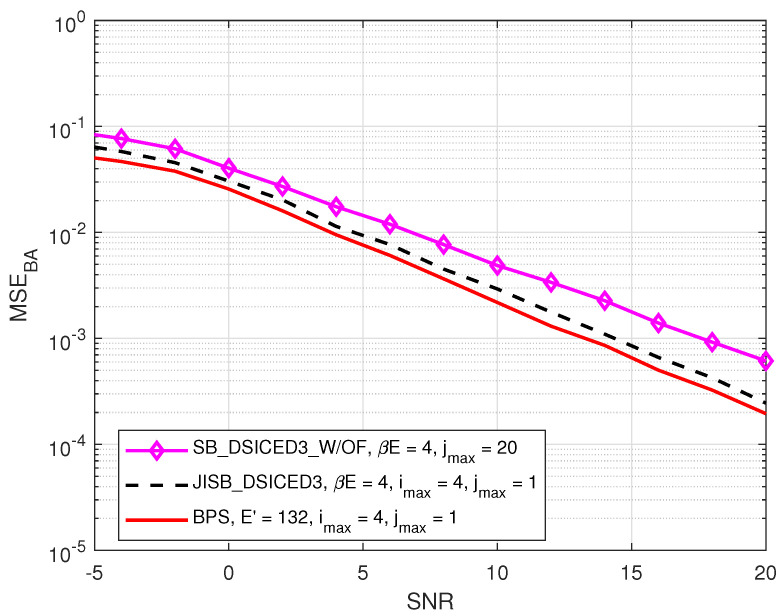
$MSE_{BA}$ versus SNR, $p_{B}/\sigma_{w}^{2}$; R=1/2, $i_{max}=4$, $j_{max}=20$, $p_{SI}/\sigma_{w}^{2}=30$ dB, βE=4 symbols, and E=128 symbols.

Figure 5 and Figure 6 compare the MSEs of two schemes, such as JISB_DSICED3 and SB_DSICED3 _W/OF, with the best performance scheme versus the SNR, $p_{B}/\sigma_{w}^{2}$, for the SI channel and the intended channel, respectively. First of all, the results showed that the feedback scheme JISB_DSICED3 had a better performance when compared with the conventional scheme SB_DSICED3_W/OF, especially at a high SNR (SNR>5 dB). Furthermore, the semi-blind feedback scheme JIB_DSICED3 showed a slightly better result at a low SNR (SNR<5 dB), compared to the conventional scheme SB_DSICED3_W/OF due to the improvement after the feedback loops. The results also showed that the semi-blind scheme JISB_DSICED3 was nearly optimal as its performance was rather closed to that of the best performance scheme, i.e., assuming that the intended symbols are known at the receiver.

### 4.2. BER Performances: JISB _DSICED3 versus SB _DSICED3 _W/OF

**Figure 7:**
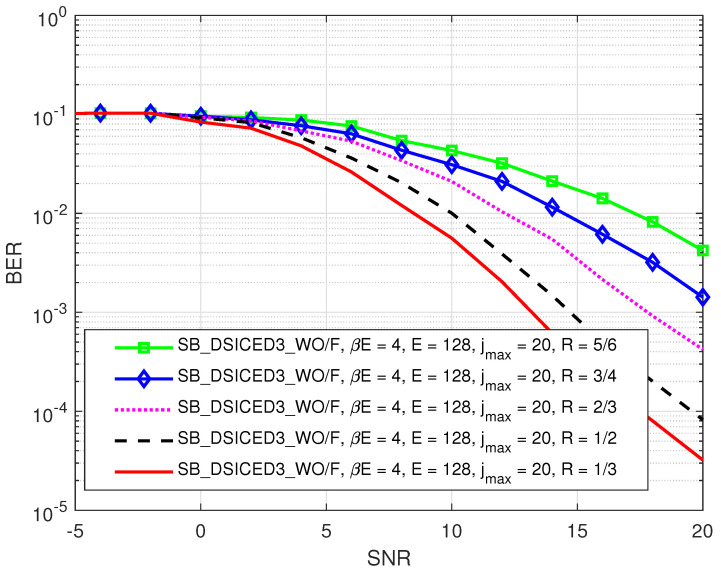
BER versus SNR, $p_{B}/\sigma_{w}^{2}$ for different code rates *R* of the SB_DSICED3_W/OF scheme; $j_{max}=20$, $p_{SI}/\sigma_{w}^{2}=30$ dB, βE=4 symbols, and E=128 symbols.

**Figure 8:**
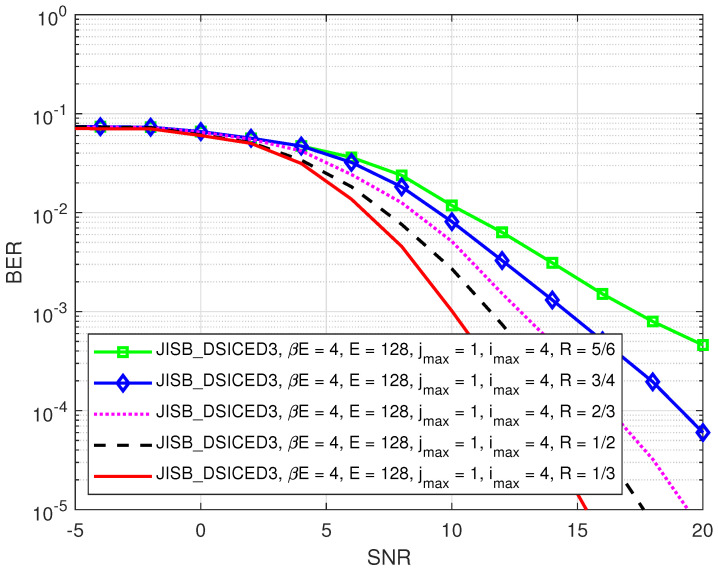
BER versus SNR, $p_{B}/\sigma_{w}^{2}$ for different code rates *R* of the JISB_DSICED3 scheme; $i_{max}=4$, $j_{max}=1$, $p_{SI}/\sigma_{w}^{2}=30$ dB, βE=4 symbols, and E=128 symbols.

First of all, the impact of different code rates such as (R∈{1/3,1/2,2/3,3/4,5/6}) on the BER performance of SB_DSICED3_W/OF and JISB_DSICED3 schemes is shown in Figure 7 and Figure 8, respectively. It can be seen that the relative behaviour of the algorithm with feedback JISB_DSICED3 with respect to the code rate behaved in the same way as the conventional algorithm without feedback SB_DSICED3_W/OF, that is when the code rate R increased, this led to a decrease in the BER performance. Therefore, the code rate should be chosen carefully based on the purposes and applications. Because the code rates can lead to either a significant loss in terms of performance (code rate R∈{2/3,3/4,5/6}) or too great a loss of throughput (code rate R=1/3,1/2), for the rest of this paper, the code rate was fixed at 1/2 as a particular example in order to illustrate the out-performance of the proposed with feedback algorithm JISB_DSICED3 over the conventional without feedback algorithm SB_DSICED3_W/OF.

**Figure 9:**
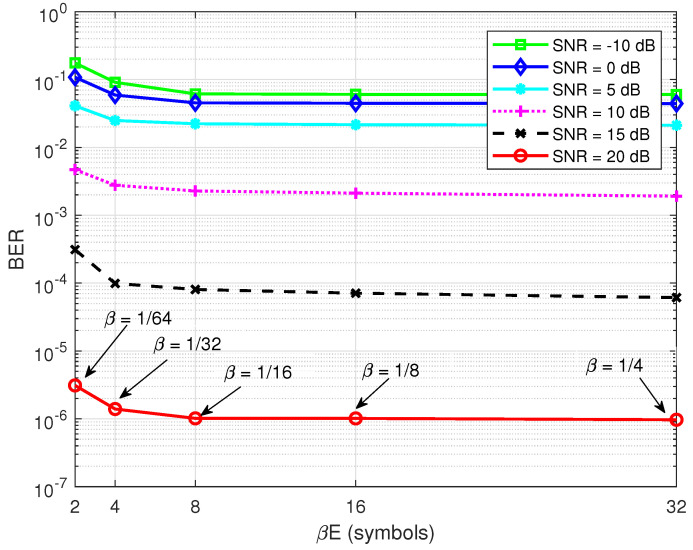
BER versus βE; R=1/2, $i_{max}=4$, $p_{SI}/\sigma_{w}^{2}=30$ dB, and E=128 symbols.

Figure 9 shows the BER of the semi-blind scheme versus the number of pilot symbols βE for different values of the SNR, $p_{B}/\sigma_{w}^{2}$, where $p_{SI}/\sigma_{w}^{2}=30$ dB and E=128 symbols. It can be seen that only a minimum of four pilot symbols (βE=4 symbols or β=1/32) was needed for the semi-blind channel estimation to achieve the saturation level. Thus, the minimum required pilot symbols made the semi-blind scheme favourable in practical implementations for short-packet transmission.

**Figure 10:**
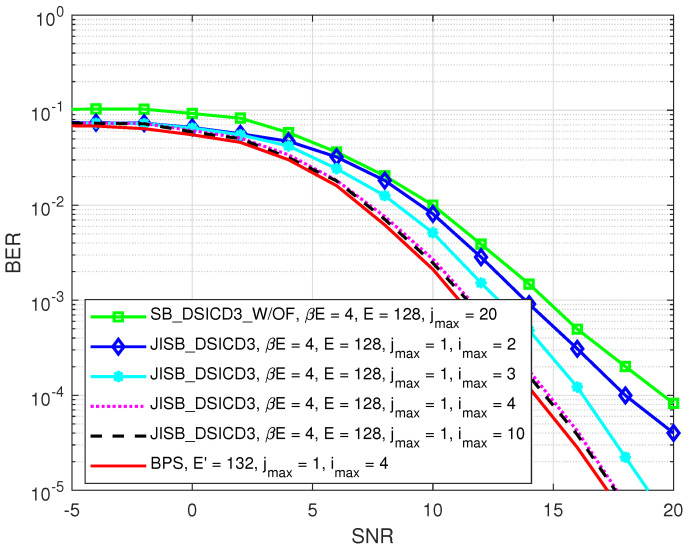
BER versus SNR, $p_{B}/\sigma_{w}^{2}$ for different values of $i_{max}$; R=1/2, $p_{SI}/\sigma_{w}^{2}=30$ dB, βE=4 symbols, and E=128 symbols.

Figure 10 illustrates the BER performance of the semi-blind scheme JISB_DSICED3 versus the SNR, $p_{B}/\sigma_{w}^{2}$, for different maximum numbers of joint iterations $i_{max}$. A BPS scheme for the BER was also calculated, which was achieved by using the best channel estimation of the SI channel and intended channel in Figure 5 and Figure 6, respectively, for the SI cancellation and SPA decoding processes with one iteration ($j_{max}=1$). It can be seen that the system achieved the convergence level when $i_{max}=4$, and the BER of the proposed JISB_DSICED3 scheme was also close to that of the lower bound (the BPS scheme).

**Figure 11:**
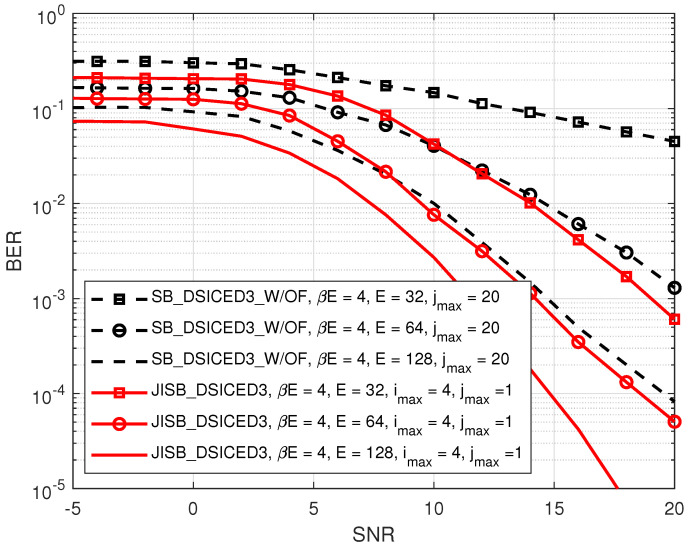
BER versus SNR, $p_{B}/\sigma_{w}^{2}$; R=1/2, βE=4, and $p_{SI}/\sigma_{w}^{2}=30$ dB.

Figure 11 compares the BERs of the semi-blind scheme JISB_DSICED3 and the conventional semi-blind without feedback scheme SB_DSICED3_W/OF versus the SNR, $p_{B}/\sigma_{w}^{2}$, for different values of E∈{32,64,128} symbols, βE=4 symbols, and $p_{SI}/\sigma_{w}^{2}=30$ dB. It can be seen that, at a low SNR (≤5 dB), the BER of the JISB_DSICED3 scheme was slightly lower than that of the SB_DSICED3_W/OF scheme regardless of the total number of transmitted symbols. However, at a high SNR (≥5 dB), the semi-blind scheme JISB_DSICED3 showed its out-performance with faster convergence. This was due to the use of a minimum of four pilot symbols, which were added to the information sequence, for better performance in the semi-blind scheme.

**Figure 12:**
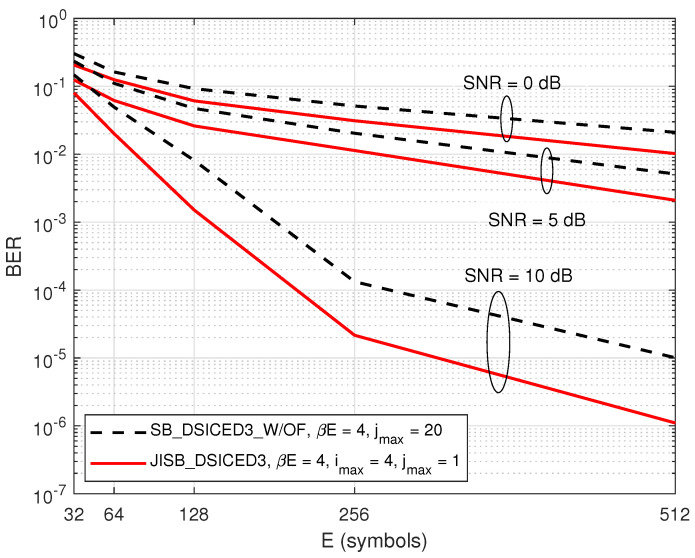
BER versus E; R=1/2, βE=4, $i_{max}=4$, and $p_{SI}/\sigma_{w}^{2}=30$ dB.

Last but not least, Figure 12 shows the BER performance versus different values of *E* symbols, where βE=4 symbols. The result indicated that the semi-blind scheme JISB_DSICED3 had better performance than the conventional semi-blind without feedback scheme SB_DSICED3_W/OF, regardless of the total number of transmitted symbols and the SNR level. It also showed that when the number of symbols *E* and the value of SNR increased, the gaps between the two schemes were bigger, which was shown clearly when SNR=10 dB. This was due to the advantage of having known pilot symbols and the feedback loops. Therefore, this indicated that the semi-blind scheme JISB_DSICED3 is an optimum solution for not only short-packet transmission, but also for a low region of the SNR, which are the operation characteristics of IoT transmission and green communication.

### 4.3. Comparison of the Processing Time and Computational Complexity

In this section, we compare the processing time and computational complexity of the proposed semi-blind scheme JISB_DSICED3 and the conventional without feedback scheme SB_DSICED3_W/OF. The processing time is an important factor for performance evaluation since it quantifies the effectiveness of the algorithm, especially in 5G short-packet transmissions and IoT applications.

In this case, a computer with the hardware configuration of Intel (R) Core (TM) I5-10500 CPU @ 3.10 GHz (12 CPUs) and a memory of 16 GB of RAM was used with MATLAB Version 2020b. For the simulation parameters, we set $p_{SI}/\sigma_{w}^{2}=30$ dB, $10^{6}$ transmission frames, E=128 symbols, β=1/32 (βE=4 symbols), $i_{max}=4$, and $j_{max}=1$ for the semi-blind scheme JISB_DSICED3 and $j_{max}=20$ for the semi-blind without feedback scheme SB_DSICED3_W/OF. Because we fixed the same value of the maximum number of decoding iterations for all levels of the SNR, the processing time was nearly the same. Therefore, this configuration was used to calculate the processing time to achieve the MSE and BER at the particular SNR level, $p_{B}/\sigma_{w}^{2}=10$ dB. Based on the results in Table 3, it was observed that the semi-blind scheme JISB_DSICED3 could significantly reduce the processing time and showed the fastest result because temporary decoding and encoding were not required in the feedback loop and the number of iterations in the SPA decoding process was also reduced, which took less roughly 10-times that compared to the semi-blind without feedback scheme SB_DSICED3_W/OF. This was due to the semi-blind scheme only needing one decoding iteration ($j_{max}=1$) to obtain a good result when achieving the best value of the channel estimations, while the semi-blind without feedback scheme SB_DSICED3_W/OF needed up to 20 iterations ($j_{max}=20$) to converge and to reach the saturation floor.

Moreover, the computational complexity of the two schemes was computed based on the summation of the number of computations in the operations including additions/subtractions, multiplications/divisions, and XOR operations based on [40,55,56,57,58,59]. Because of the identity and symmetry at the transmitter side, this paper only considered calculating the total number of computations at the receiver side. The formulas for calculating the relative number of computations for each operation are summarised in detail in Table 4, where ${\bar{u}}_{v},{\bar{u}}_{c}$ denote the average degree of the variable nodes and the average degree of the check nodes of the parity check matrix H, respectively.

**Figure 13:**
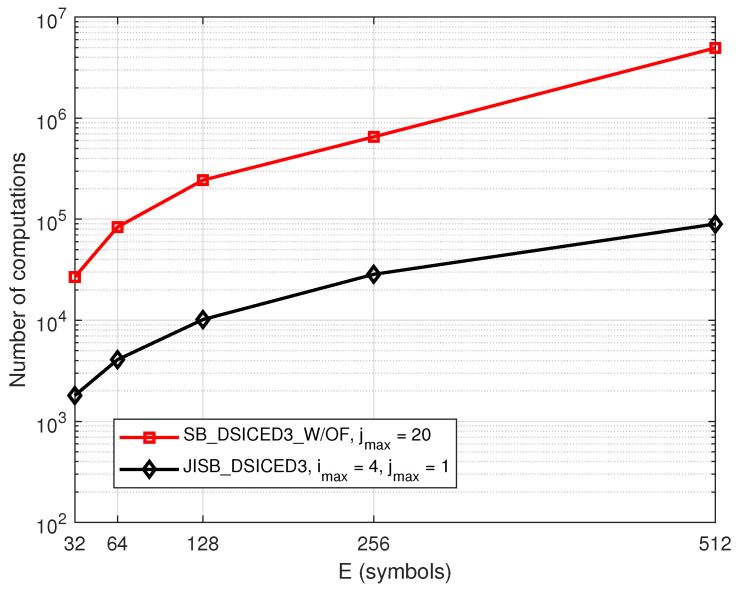
Number of computations versus *E*; R=1/2, βE=4, $p_{B}/\sigma_{w}^{2}=10$ dB, $p_{SI}/\sigma_{w}^{2}=30$ dB.

Figure 13 shows the number of computations versus various values of the symbols *E*, which was used to calculate the total number of computations to obtain the MSE and BER at the particular SNR level, $p_{B}/\sigma_{w}^{2}=10$ dB. For the simulation parameters, we set βE=4 symbols, $p_{SI}/\sigma_{w}^{2}=30$ dB, $10^{6}$ transmission frames, $i_{max}=4$, and $j_{max}=1$ for the proposed feedback schemes JISB_DSICED3 and $j_{max}=20$ for the conventional without feedback scheme SB_DSICED3_W/OF. The result indicated that the proposed feedback scheme JISB_DSICED3 required less cost for completing the computation than the conventional without feedback scheme SB_DSICED3_W/OF.

Therefore, this result emphasises the practical implementation of the scheme in 5G short-packet transmissions, especially in IoT transmissions and green communications with low power consumption.

## 5. Conclusions

This paper proposed a joint iterative semi-blind SI cancellation and intended channel estimation in 5G QC-LDPC-encoded short-packet FD transmissions, via the feedback of the known pilot symbols, named the JISB_DSICED3 scheme. The innovation of the proposed algorithm was taking advantage of iterative algorithms to design simultaneous SI cancellation and intended channel estimation in order to efficiently cancel the SI component and improve the simultaneous channel estimation in the next iterations. This semi-blind algorithm adds only a minimum of four pilot symbols to the information symbols while not requiring the feedback of temporary decoded messages. The numerical results showed that the proposed semi-blind algorithm JISB_DSICED3 was nearly optimal and efficiently increased the performance of the MSE and BER. The significant processing time and computational complexity reduction of the semi-blind feedback algorithm JISB_DSICED3 were impressive as it only required the feedback of the channel estimate for $i_{max}=4$ iterations and only one ($j_{max}=1$) decoding iteration where the decoding algorithm had a prohibitive computation cost. All these results indicate the efficient use of this semi-blind feedback algorithm, especially since the use or the insertion of these pilot symbols did not in practice really lead to a loss of the data rate because they were already generally required for time and frequency synchronisation.

## 6. Future Works

There are many interesting factors that still remain and should be examined further in FD short-packet transmission and channel coding schemes, especially in the physical layer security area. The impacts of a higher order of modulation in FD short-packet transmissions need to be considered. Therefore, it is possible to expand this work to the non-binary LDPC codes, which are used to overcome the weakness of the binary codes in short code lengths and higher orders of modulation such as 16-QAM or 64-QAM [60]. In the near future, a Software-Defined Radio (SDR) implementation of the proposed algorithm will be developed in realistic transmission scenarios in order to evaluate its performance on real signals, especially for IoT applications and green communications. Furthermore, we would like also to evaluate the proposed algorithm combined with self-jamming techniques in the physical layer security area for FD short-packet transmissions with the presence of an eavesdropper. Last but not least, the theoretical and analytical approach for both the Cramér–Rao Lower Bounds (CRLBs) for channel estimation and also the lower bound of the BER will be considered in the near future.

## Figures and Tables

**Table 1:** List of notations.

Notations	Meaning
*K*	Information length
*N*	Codeword length
*R*	Code rate
*M*	Modulation order
*E*	Frame length after modulation
$E^{\prime }$	Frame length after adding pilot symbols
β	Pilot symbol coefficient
$h_{XY}$	Channel gain vector between X and Y
$h_{XX}$	Self-interference channel gain vector
x[k]	*k*-th bit of signal vector x in the bit domain
x[n]	*n*-th symbol of signal vector x in the discrete time domain
$x^{\prime }\left[n\right]$	*n*-th symbol of concatenation signal vector x with pilot symbols in the
	discrete time domain
$x^{\prime }\left(t\right)$	Signal *x*′ in the continuous time domain
$\hat{x}$	Estimation value of x
$\tilde{x}$	Residual value of x
∗	Convolution operator
λ	Forget factor of the RLS algorithm
*i*	Index of joint iterative iterations
*j*	Index of 5G QC-LDPC decoding iterations
${\bar{u}}_{v}$	Average degree of the variable nodes
${\bar{u}}_{c}$	Average degree of the check nodes

**Table 2:** Simulation specifications.

Parameter	Value
Codeword length (*N*)	64, 128, 256, 512, 1024
Information length (*K*)	32, 64, 128, 256, 512
Code rate (*R*)	1/3, 1/2, 2/3, 3/4, 5/6
Number of transmission frames	1,000,000
Modulation scheme	QPSK (M=4)
SI channel taps	3
Intended channel taps	4
Forget factor λ	0.999
Index of joint iterations in JISB_DSICED3 scheme	$i_{max}=4,j_{max}=1$
Index of iteration of SPA decoding in SB_DSICED3_W/OF	$j_{max}=20$

**Table 3:** Processing time.

Algorithm	Processing Time (in min)	Ratio Respected to (SB_DSICED3_W/OF)
SB_DSICED3_W/OF scheme	475.7	1
JISB_DSICED3 scheme	48.1	0.101

**Table 4:** Summary of the number of computations.

Operation	Number of Computations
Demodulation	O(N)
De-Interleaver	O(N)
SPA decoding	$j_{max}.\left(\left(2.N.{\bar{u}}_{v}+\left(N-K\right)\left(3.{\bar{u}}_{c}-1\right)\right)\right)$
RLS algorithm	$O({E^{\prime }}^{2})$

## Data Availability

The data presented in this study are available upon request from the corresponding author.

## Abbreviations

The following abbreviations are used in this manuscript:

3GPP	3rd Generation Partnership Project
5G	Fifth Generation
ADC	Analogue-to-Digital Converter
AF	Amplify-and-Forward
BER	Bit Error Rate
BPS	Best Performance Scheme
CRLBs	Cramér–Rao Lower Bounds
CFO	Carrier Frequency Offset
DAC	Digital-to-Analogue Converter
DSIC	Digital Self-interference Cancellation
FD	Full-Duplex
FDD	Frequency Division Duplex
HD	Half-Duplex
ICA	Independent Component Analysis
IoT	Internet of Things
ITU	International Telecommunication Union
JISB_DSICED	Joint Iterative Semi-Blind Digital Self-Interference Cancellation,
	Equalisation, Demodulation, De-interleaving, and Decoding
LLR	Log Likelihood Ratio
LMS	Least Mean Squares
LoS	Line-of-Sight
mM2M	massive Machine-to-Machine
mMTC	massive Machine-Type Communications
MSE	Mean-Squared Error
NLMS	Normalised Least Mean Squares
NLoS	Non Line-of-Sight
PLS	Physical Layer Security
QC-LDPC	Quasi-Cyclic Low-Density Parity Check
QPSK	Quadrature Phase Shift Keying
RF	Radio Frequency
RLS	Recursive Least Squares
S-MMSE	Semi-blind Minimum Mean-Squared Error
SB_DSICED3_W/OF	Semi-Blind Digital Self-Interference Cancellation, Equalisation,
	Demodulation, De-interleaving, and Decoding Without Feedback
SDR	Software-Defined Radio
SI	Self-Interference
SPA	Sum Product Algorithm
TDD	Time Division Duplex
uRLLC	ultra-Reliable Low-Latency Communications
